# Chronotropic Incompetence after Heart Transplantation Is Associated with Increased Mortality and Decreased Functional Capacity

**DOI:** 10.3390/jcm12103487

**Published:** 2023-05-16

**Authors:** Robert S. Zhang, Thomas C. Hanff, Yuhui Zhang, Michael V. Genuardi, Carli J. Peters, Allison Levin, Maria Molina, Rhondalyn C. McLean, Jeremy A. Mazurek, Payman Zamani, Monique S. Tanna, Joyce Wald, Pasquale Santangeli, Pavan Atluri, Lee R. Goldberg, Edo Y. Birati

**Affiliations:** 1 Division of Cardiovascular Medicine, NYU Langone Health, New York, NY 10016, USA; robert.zhang@nyulangone.org (R.S.Z.); carli.peters@pennmedicine.upenn.edu (C.J.P.); 2 Department of Medicine, Perelman School of Medicine at the University of Pennsylvania, Philadelphia, PA 19123, USA; thomas.hanff2@pennmedicine.upenn.edu (T.C.H.); michael.genuardi@pennmedicine.upenn.edu (M.V.G.); allison.levin@duke.edu (A.L.); rhondalyn.mclean@pennmedicine.upenn.edu (R.C.M.); jeremy.mazurek@pennmedicine.upenn.edu (J.A.M.); payman.zamani@pennmedicine.upenn.edu (P.Z.); monique.tanna@pennmedicine.upenn.edu (M.S.T.); joyce.wald@pennmedicine.upenn.edu (J.W.); pasquale.santangeli@pennmedicine.upenn.edu (P.S.); lee.goldberg@pennmedicine.upenn.edu (L.R.G.); 3 Division of Cardiology, Perelman School of Medicine at the University of Pennsylvania, Philadelphia, PA 19123, USA; maria.molina@pennmedicine.upenn.edu; 4 Fuwai Hospital, Peking Union Medical College, Beijing 100005, China; yuhuizhangjoy@126.com; 5 Department of Cardiothoracic Surgery, Perelman School of Medicine, University of Pennsylvania, Philadelphia, PA 19123, USA; pavan.atluri@pennmedicine.upenn.edu; 6 The Lydia and Carol Kittner, Lea and Banjamin Davidai Division of Cardiovascular Medicine and Surgery, Padeh-Poriya Medical Center, Tiberias 1528001, Israel

**Keywords:** transplant, exercise, survival, prognosis, heart rate, heart rate response

## Abstract

Introduction: The contribution of chronotropic incompetence to reduced exercise tolerance after a heart transplant is well known, but its role as a prognostic marker of post-transplant mortality is unclear. The aim of this study is to examine the relationship between post-transplant heart rate response (HRR) and survival. Methods: We performed a retrospective analysis of all adult heart transplant recipients at the University of Pennsylvania between the years 2000 and 2011 who underwent a cardiopulmonary exercise test (CPET) within a year of transplant. Follow-up time and survival status were observed through October 2019, using data merged from the Penn Transplant Institute. HRR was calculated by subtracting the resting HR from the peak exercise HR. The association between HRR and mortality was analyzed using Cox proportional hazard models and Kaplan–Meier analysis. The optimal cut-off point for HRR was generated by Harrell’s C statistic. Patients with submaximal exercise tests were excluded, defined by a respiratory exchange ratio (RER) cut-off of 1.05. Results: Of 277 patients with CPETs performed within a year post-transplant, 67 were excluded for submaximal exercise. In the 210 included patients, the mean follow-up time was 10.9 years (Interquartile range (IQR) 7.8–14). Resting HR and peak HR did not significantly impact mortality after adjusting for covariates. In a multivariable linear regression analysis, each 10-beat increase in heart rate response was associated with a 1.3 mL/kg/min increase in peak V_O_2__ and a 48 s increase in the total exercise time. Each beat/min increase in HRR was associated with a 3% reduction in the hazard of mortality (HR 0.97; 95% CI 0.96–0.99, *p* = 0.002). Using the optimal cut-off point generated by Harrell’s C statistic, survival was significantly higher in patients with an HRR > 35 beats/min compared to those with an HRR < 35 beats/min (log rank *p* = 0.0012). Conclusion: In heart transplant patients, a low HRR is associated with increased all-cause mortality and decreased exercise capacity. Additional studies are needed to validate whether targeting HRR in cardiac rehabilitation may improve outcomes.

## 1. Introduction

Orthotopic heart transplantation (OHT) is a well-established treatment for patients with advanced heart failure, associated with significant improvements in both survival and quality of life [1]. While post-transplant exercise capacity is increased compared to pre-transplant, it remains lower than normal for age-matched controls [2]. One of the driving forces behind this phenomenon is an abnormal chronotropic response to exercise, known as chronotropic incompetence (CI), due in part to the cardiac denervation that occurs during heart transplantation. CI is defined as the heart’s inability to appropriately increase its rate to increased activity or demand, although a standardized definition is lacking [3]. Notably, the widely utilized formula described by Astrand et al. for age-predicted maximal heart rate (APMHR = 220-age) was derived from a healthy population with wide confidence intervals around this estimation [4]. Thus, it is unlikely to accurately reflect the maximal predicted heart rate (HR) in heart transplant patients with autonomic denervation. An alternative approach is to assess chronotropic response by comparing peak HR at maximal exertion during the exercise test to the resting heart rate, otherwise known as heart rate response (HRR). HRR allows for the assessment of the dynamic HR range, taking baseline HR into account rather than relying on peak HR alone. While chronotropic incompetence after heart transplantation is expected and is known to affect exercise capacity, it is unclear how HRR in the transplant population affects mortality [5,6]. In a previous study, our group demonstrated a positive association between post-transplant peak oxygen consumption (peak V_O_2__) and long term survival [7]. The aim of this study is to evaluate the association between post-transplant heart rate response and survival, and to describe the association of heart rate response with exercise capacity.

## 2. Methods

### 2.1. Design and Participants

We performed a retrospective analysis of all patients aged ≥18 years who underwent heart transplantation between 2000 and 2011 at the University of Pennsylvania. Survival data were collected through October 2019 using data merged from the Penn Transplant Institute. Patients who underwent re-transplantation or multi-organ transplant were excluded. This era was selected due to a clinical protocol at the time in which all patients underwent a post-transplant cardiopulmonary exercise test (CPET) within one year of transplant if clinically able. Patients who underwent CPET outside of their first post-transplant year were excluded. Patients with permanent pacemakers were also excluded. Pre- and post-transplant clinical data, including baseline demographics, CPET results, medications, laboratory values at the time of CPET, and post-transplant echocardiogram, were obtained from patients’ electronic medical records. This study was approved by the University of Pennsylvania Institutional Review Board.

### 2.2. Cardiopulmonary Exercise Metrics

All patients performed a symptom-limited treadmill CPET according to our standard clinical practice, up to the maximal volitional effort, as described in previous studies. Studies were interpreted by an advanced heart failure cardiologist. Prior to exercise, resting ECG and vital signs were recorded, including heart rate, as well as baseline respiratory mechanics and maximal voluntary ventilation (MVV). Breath-by-breath expired gases were obtained to estimate minute ventilation (V_E_), carbon dioxide production (V_CO_2__), and V_O_2__, which were displayed as 10 s averages. Vital signs were obtained multiple times throughout the CPET, including at peak exercise. Peak V_O_2__ and the respiratory exchange ratio (RER) were determined as the highest 10-s averaged samples obtained during the exercise test. The RER was determined from the ratio of V_CO_2__ to V_O_2__. The maximal volitional exercise was determined using a peak RER ≥ 1.05, based on guideline recommendation [8,9]. O_2_ pulse was calculated as the ratio of unindexed V_O_2__ to heart rate. The V-slope method was used to determine the ventilatory threshold (VT). Ventilatory equivalents for carbon dioxide (V_E_V_CO_2__) were measured at VT and at peak exercise, and end-tidal P_CO_2__ was measured at peak exercise (PET_CO_2__). Breathing reserve was calculated as the difference between MVV and maximum V_E_ as a percent of MVV (i.e., [MVV − V_E__Max_]/MVV × 100%). HRR was calculated by subtracting the resting HR from the peak HR. Heart rate reserve (which is distinct from the heart rate response, HRR) was calculated by dividing HRR by the difference between resting HR and APMHR according to the following formula: Heart Rate Reserve = ([peak HR − resting HR]/[APMHR − resting HR] × 100) [8]. Only studies demonstrating evidence of maximal effort were used in the analyses. The first post-transplant transthoracic echocardiogram obtained within one year of the CPET was used to compare peak V_O_2__ to the left ventricular ejection fraction.

### 2.3. Statistical Analysis

Continuous variables were presented as mean ± standard deviation (SD) or median with interquartile range (IQR) for skewed data. Categorical data were expressed as frequency and proportions and compared using Fisher’s exact test. Baseline characteristics in patients who had an HRR above and below the sample median were compared using the Student’s *t*-test or the Wilcox rank-sum, as appropriate.

To evaluate the association between HRR and all-cause mortality, Kaplan–Meier survival curves were generated for the two groups based on HRR above and below an optimal cut-off point generated by Harrell’s C statistic. The survival endpoint was survival free from re-transplantation. The log rank test was used to compare the survival between the two groups. The relationship between HRR and all-cause mortality at follow-up was assessed using a Cox proportional hazards model, where HRR was entered as a continuous variable. The model was adjusted for covariates measured prior to CPET, including age, gender, race, BMI, beta-blocker use, hemoglobin at the time of CPET, a history of chronic obstructive pulmonary disease (COPD), and post-transplant ejection fraction (EF). Similar models were developed to examine the relationship between peak HR and mortality. Schoenfeld residuals were used to test the proportional hazard assumption of the Cox model.

To examine the relationship between HRR and peak V_O_2__, we fit a multivariable linear regression model with HRR as the independent variable, adjusting for the same clinical and laboratory covariates as above. We repeated this method to assess the relationship between HRR and exercise time. All tests were considered significant at a two-sided alpha level < 0.05. The rate of missing data for each covariate did not exceed 15%. Missing values for the covariates in each model were considered missing at random and filled in via multiple imputations with chained equations based on twenty imputations using Rubin’s combination rules. All analyses were performed in Stata version 15 (College Station, TX, USA, StataCorp LLC).

## 3. Results

A total of 418 patients underwent heart transplantation at our institution. Of these patients, 277 were included after removing 115 patients with no CPET, 23 with CPET > 1-year post-transplant, and three patients who underwent re-transplantation. Among these 277 patients, 67 had submaximal CPETs and were excluded from the analysis. In the 210 patients with a maximal effort CPET within one year of a first-time heart transplant, the mean follow-up time was 10.9 years with an interquartile range of 7.8 and 14 years.

Baseline demographics and CPET parameters in patients above and below an HRR of 35 beats/min are displayed in Table 1. Patients with a lower HRR were more likely to have COPD (12% vs. 2% *p* = 0.011) but otherwise had similar distributions of age, race, gender, and other pre-transplant comorbidities. Notably, there was no difference in post-transplant EF and beta-blocker use at the time of CPET.

Several differences were present between the two groups in regard to CPET metrics. Patients with an HRR above 35 beats/min had a higher peak heart rate (110.3 vs. 134.9 bpm, *p* < 0.001), higher peak systolic blood pressure (147 vs. 157 mmHg (*p* < 0.001), higher peak V_O_2__ (14.5 vs. 17.4 mL/kg/min, *p* < 0.001), longer total exercise time (7.7 vs. 9.3 min, *p* < 0.001), higher maximum voluntary ventilation (102.6 vs. 113.4 L, *p* = 0.011), higher heart rate reserve (27 vs. 59%, *p* < 0.001), and a lower V_E_/V_CO_2__ (40.5 vs. 37.9 *p* = 0.008).

### Heart Rate Response and Survival

**Figure 1:**
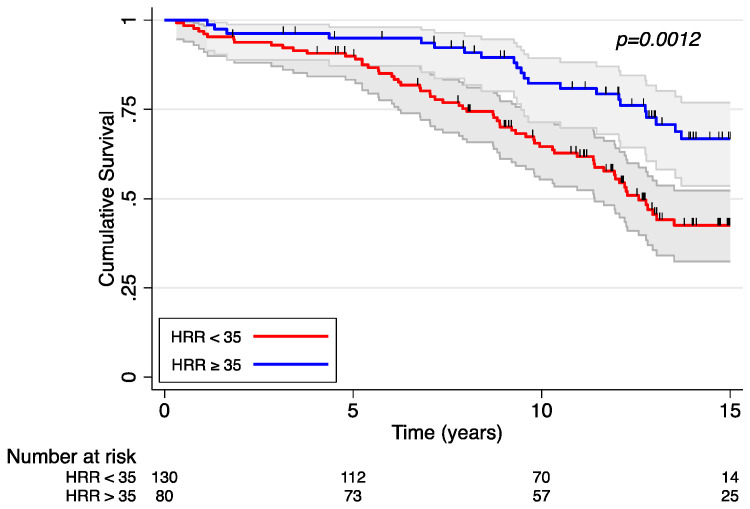
Kaplan–Meier survival curve stratified by heart rate response.

**Figure 2:**
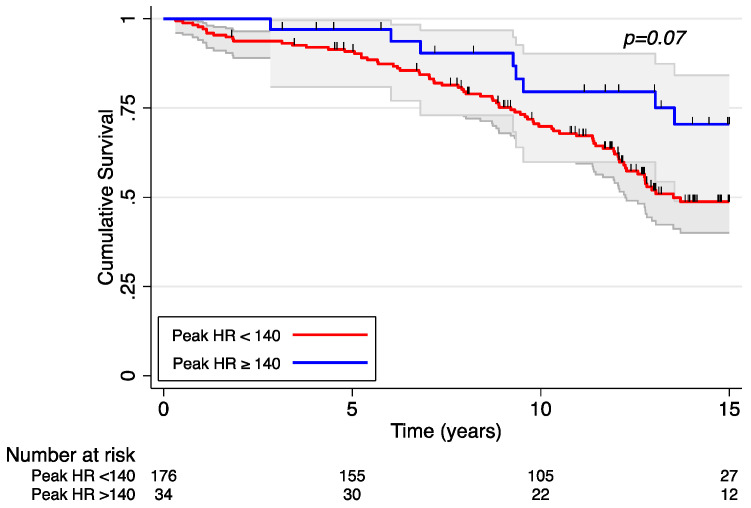
Kaplan–Meier survival curve stratified by peak heart rate.

Of the 210 patients in this analysis, a total of 88 patients died over the course of follow-up over 18 years. HRR was independently associated with mortality before and after adjustment for covariates (Table 2). Each beat/min increase in HRR was associated with a 3% reduction in the hazard of mortality (HR 0.97; 95% CI 0.96–0.99, *p* = 0.002). In Figure 1, the Kaplan–Meier curve demonstrates a significantly increased survival in patients with an HRR ≥ 35 beats/min compared to those with an HRR < 35 beats/min across 15 years of follow-up. The 10-year survival rate was 64% among those in the lower HRR group and 82% in the higher HRR group. Although peak heart rate was associated with decreased mortality in the univariable analysis, it was no longer associated with mortality when adjusted for covariates. Figure 2 demonstrates a Kaplan–Meier survival curve stratified by peak HR above and below 140 beats/min. Resting heart rate was not associated with mortality in our cohort, even when including patients who had submaximal effort.

**Figure 3:**
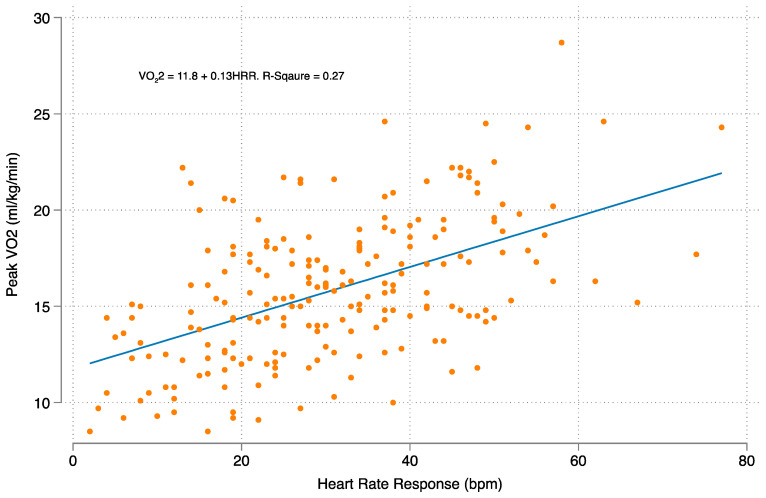
The relationship between heart rate response and peak V_O_2__.

**Figure 4:**
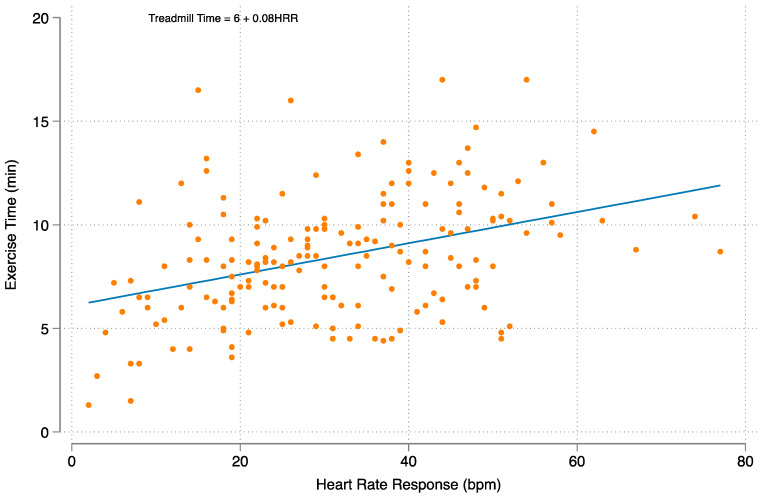
The relationship between heart rate response and exercise time.

Linear regression models were constructed to examine the relationship between HRR, peak V_O_2__, and treadmill time (Figure 3 and Figure 4). In a multivariable linear regression analysis, each 10-beat increase in heart rate response was associated with a 1.3 mL/kg/min increase in peak V_O_2__ and a 48 s increase in the total exercise time.

## 4. Discussion

Following a heart transplant, patients commonly have a persistently lower exercise capacity when compared with age-matched controls [10]. CI is a key contributor to this reduced exercise capacity [11,12,13]. However, the association between CI and mortality in this population has not been established, in part because no clear definition of chronotropic incompetence exists in patients after heart transplantation. The traditional formula of age-predicted maximal heart rate is unlikely to apply to these patients with a denervated orthotopic heart [4]. The HRR (i.e., the difference between peak HR at maximal exertion to resting heart rate) may better reflect CI in patients after OHT. In this study, we observed a significant association between higher HRR and longer post-transplant survival free from re-transplantation. Using the endpoint of survival, we then derived a threshold for HRR that optimized the calibration of the survival model.

To our knowledge, this is the first study that has demonstrated the relationship between HRR and long-term post-transplant mortality. In our large cohort of OHT patients, we found that an attenuated heart rate response to exercise is associated with increased all-cause mortality over more than a decade of follow-up. More specifically, a heart rate response of less than 35 beats/min provides a useful threshold that optimizes the prediction of all-cause mortality compared. The association between HRR and survival held true when adjusting for demographic factors and relevant comorbidities. The differences in HRR in our cohort were primarily attributed to differences in peak heart rate given the similar baseline resting heart rates. Notably, resting heart rate was not associated with the risk of mortality, even when including patients who had submaximal exercise stress tests. This is contrary to some of the current literature [14,15]. This may be due to the different time points when post-transplant resting heart rates were evaluated.

It is well known that chronotropic incompetence is associated with increased all-cause and cardiovascular mortality in heart failure and healthy individuals [8,16,17,18]. This has not been thoroughly explored in post-transplant population, where the denervated heart responds differently and may not reflect any underlying structural changes in the heart predictive of survival. Our study suggests that poor heart rate response with exercise is also associated with increased mortality in heart transplant patients.

The underlying mechanisms to explain how a higher HRR contributes to improved survival in heart transplant patients is unclear but is likely a multifactorial phenomenon. The natural history of HT patients involves the gradual autonomic reinnervation of the cardiac allograft, which leads to improved HRR to exercise [19]. The significant variability of sympathetic reinnervation from patient to patient may be contributing. HRR may contribute to a reduction in mortality as a marker of exercise capacity and overall physical fitness. Heart rate response correlates with exercise capacity in the normal population, and the degree of physical fitness has been shown to be predictive of cardiac risk and mortality [3,20]. In our cohort, we demonstrated that HRR is associated with both peak V_O_2__ and total exercise time, as also seen in other studies [12]. Given the strong relationship between HRR and peak V_O_2__, it is possible that skeletal muscle metabolic abnormalities, pre-transplant sarcopenia and frailty, and deconditioning may persist and contribute to overall exercise limitation [21].

Our findings have important clinical implications. First, this study demonstrates that the assessment of HRR carries important prognostic value in the heart transplant population. Importantly, HRR is a potentially modifiable risk factor that may be improved by endurance and exercise training. Additional studies are needed to elucidate the mechanism of the increased mortality risk in patients with a lower HRR. Lastly, HRR may be a more accessible and inexpensive tool for risk stratification in place of peak V_O_2__, which was recently found to be a predictor of mortality in the heart transplant population [7].

### Limitations

This is a single-center study, which limits its generalizability. The cut-off values for heart rate response were based on an optimization algorithm and will need to be validated in other data sets. Patients who did not undergo CPET were excluded from our study, which introduces selection bias as these individuals are more likely to have died without undergoing CPET or be too ill to undergo CPET. Patients without CPET had higher mortality than patients with CPETs; thus, our study likely underestimates the overall mortality of our OHT cohort. Although we included only patients who performed a CPET within a year, the time at which each patient obtained a CPET varied. This variability may be subject to other confounding factors, such as incomplete recovery from surgery, less time to recover from pretransplant sarcopenia, and frailty. In an attempt to limit such confounding, we only included patients with a respiratory exchange ratio (RER) greater than 1.05 in the study and did not include patients with CPETs performed more than one-year post-transplant. However, residual confounding cannot be entirely ruled out. This study is also limited by its retrospective nature, which increases the possibility of comorbidity misclassification.

## 5. Conclusions

In heart transplant patients, a low HRR is associated with increased all-cause mortality. HRR may be a more accessible and inexpensive tool for risk stratification when peak V_O_2__ cannot be obtained. Future studies are needed to evaluate whether the reduced HRR is only a sign of frailty and lack of fitness and whether strategies can be implemented to improve the long-term outcomes of this patient population.

## Figures and Tables

**Table 1:** Baseline characteristics of patients with a heart rate response (HRR) above and below 35 beats/min.

	HRR < 35 Beats/min	HRR ≥ 35 Beats/min	*p*-Value
Baseline Characteristics	N = 130	N = 80	
Age at CPET, years	52.5 (12.1)	52.2 (13.2)	0.86
Sex, male	108 (83.1%)	68 (85.0%)	0.71
Race			0.30
Caucasian	104 (80.0%)	57 (71.2%)	
African American	20 (15.4%)	19 (23.8%)	
Other	6 (4.6%)	4 (5.0%)	
Pre-Transplant BMI, kg/m^2^	27.1 (4.6)	28.8 (5.1)	0.018
BMI, kg/m^2^	27.0 (4.7)	28.6 (4.9)	0.021
Diabetes Pre-Transplant	55 (42.3%)	27 (34.2%)	0.24
COPD Pre-Transplant	12 (9.2%)	1 (1.2%)	0.020
Ischemic Cardiomyopathy	57 (53.3%)	29 (47.5%)	0.47
Hemoglobin at CPET (g/dL)	12.8 (1.8)	13.0 (1.8)	0.37
Creatinine at CPET (mg/dL)	1.5 (0.8)	1.5 (0.6)	0.86
Beta Blocker Use at CPET	25 (19.2%)	18 (23.1%)	0.51
LVEDd, cm	4.6 (0.6)	4.5 (0.5)	0.49
EF, %	65.2 (9.8)	65.1 (8.5)	0.96
Post-transplant RV dilation (% of patients)	41 (32%)	37 (46%)	0.009
Post-transplant RV dysfunction (% of patients)	28 (22%)	22 (28%)	0.23
Post-transplant PASP, mmHg	35 (7.8)	36 (7.9)	0.43
Ischemic time (hours)	3 (0.9)	3 (0.8)	0.91
CPET Parameters			
Months from transplant to CPET	3.2 (2.7)	4.0 (3.6)	0.062
Resting heart rate (bpm)	90.8 (11.1)	91.5 (12.6)	0.69
Peak heart rate (bpm)	112.4 (14.7)	137.4 (14.0)	<0.001
Resting systolic BP, mmHg	127.0 (15.6)	128.7 (15.1)	0.44
Peak systolic BP, mmHg	148 (22)	157(22)	0.003
Peak V_O_2__, ml/kg/min	14.6 (3.2)	17.8 (3.5)	<0.001
Total exercise time, min	7.7 (2.7)	9.6 (2.9)	<0.001
Respiratory exchange ratio	1.16 (0.1)	1.18 (0.1)	0.022
Ventilatory threshold L/min	0.9 (0.3)	1.0 (0.3)	<0.001
O_2_ pulse, ml/beat	11.0 (3.0)	11.5 (3.0)	0.33
Heart rate reserve (%)	29.3 (13.1)	62.8 (18.8)	<0.001
Peak respiratory exchange ratio	1.2 (0.1)	1.2 (0.1)	0.022
V_E_/V_CO_2__	40.1 (5.9)	37.9 (5.2)	0.008
Maximum voluntary ventilation, L	103.1 (27.6)	115.3 (34.6)	0.005
Breathing reserve (%)	38.2 (16.3)	33.9 (17.7)	0.081

Values are mean ± standard deviation or number (proportion). Acronyms: BMI, body mass index; BP, diastolic blood pressure; COPD, chronic obstructive pulmonary disease; CPET, cardiopulmonary exercise test; V_CO_2__, carbon dioxide output; Ve/V_CO_2__, minute ventilation/carbon dioxide production; V_O_2__, oxygen consumption.

**Table 2:** Association between heart rate response and survival in Cox proportional hazard analyses.

	Univariable Analysis		Multivariate Analysis	
	HR (95% CI)	*p*-Value	HR (95% CI)	*p*-Value
Heart rate reserve	0.99 (0.98, 1.0)	0.10		
Heart rate response	0.98 (0.97, 0.99)	0.01	0.97 (0.96, 0.99)	0.002
Peak heart rate	0.98 (0.97, 0.99)	0.02	0.98 (0.97, 1.0)	0.06
Resting heart rate	0.99 (0.98, 1.01)	0.79		
Resting heart rate *	0.99 (0.98, 1.01)	0.30		

* Including patients with submaximal effort.

## Data Availability

The data that support the findings are available on request from the first author, R.S.Z.

## Abbreviations

CPET	cardiopulmonary exercise test
HTR	heart transplant recipients
HRR	heart rate response
CI	chronotropic incompetence
APMHR	age-predicted maximum heart rate
OHT	orthotopic heart transplantation
MVV	maximum voluntary ventilation
PET_CO_2__	peak end-tidal carbon dioxide
RER	respiratory exchange ratio
V_CO_2__	carbon dioxide production
V_E_	minute ventilation
V_O_2__	oxygen consumption
VT	ventilatory threshold
